# The Influence of Material and Veneering Technique on the Marginal Fit of CAD/CAM Crowns

**DOI:** 10.3390/dj14070397

**Published:** 2026-07-01

**Authors:** Nader Abdulhameed, Jean Francois Roulet, Hind Hussein, Zahraa Mahdi, Noor Ibrahim, Taiseer Sulaiman, Emmanouil-George Tzanakakis, Panagiotis Zoidis

**Affiliations:** 1 Restorative Dental Sciences, College of Dentistry, University of Florida, Gainesville, FL 32610, USA; naderf@ufl.edu (N.A.); jroulet@ufl.edu (J.F.R.); hinds@ufl.edu (H.H.); pzoidis@ufl.edu (P.Z.); 2 Independent Dental Research Group, Chicago, IL 60563, USA; drzalrawi@gmail.com (Z.M.); noor85dentist@yahoo.com (N.I.); 3 Division of Comprehensive Oral Health, University of North Carolina, Chapel Hill, NC 27599, USA; taiseer_sulaiman@unc.edu; 4 Department of Dentistry, School of Dentistry, European University Cyprus, 1516 Nicosia, Cyprus

**Keywords:** lithium disilicate, zirconia, CAD-CAM, marginal fit, glazing, veneering

## Abstract

**Background:** There are certain disadvantages to using CAD/CAM technologies. Marginal and internal accuracy of fit is valued as one of the most important criteria for the clinical quality and success of all-ceramic crowns. The assessment of the marginal fit of lithium disilicate and zirconia CAD/CAM crowns before and after ceramic layering is crucial. **Methods:** One ideally prepared model tooth was duplicated into 64 plaster models. A standardized wax pattern for a monolithic crown and a coping were produced and used to mill 16 lithium disilicate monolithic crowns and 16 cores using a soft milling process. 16 zirconia crowns and 16 cores were also fabricated. A factorial design with (material) (lithium disilicate [E] or zirconia [Z]); (design) (monolithic, [M] or (core) [C]); and (finish) (as-produced [P] or veneered/glazed [G]) was used to create the following groups: ZMP, ZMG, ZCP, ZCG, EMP, EMG, ECP, and ECG (n = 8). The milled restorations were treated accordingly using ZirLiner, IPS e.max Ceram, and IPS e.max Glaze. The restorations were cemented to their dies, embedded in epoxy resin, and sectioned into two planes with a diamond saw. Vertical and horizontal marginal fit at the finishing line was measured in a standardized way at four locations (mesial, distal, facial, and lingual). **Results:** There were no differences, *p* > 0.05, between all Z groups; however, they had significantly wider horizontal gaps, *p* < 0.05, (116 ± 5 µm) than E groups (64 ± 13 µm). Among lithium disilicate groups, the glazed monolithic (EMG) and veneered/glazed coping (ECG) subgroups showed significantly smaller horizontal gaps (approximately 50 ± 6 µm). Statistical analysis was performed using two-way ANOVA with a significance level set at α = 0.05. **Conclusions:** Veneering techniques did not affect zirconia. Lithium disilicate had a better marginal fit than zirconia, but this was influenced by veneering techniques. Lithium disilicate veneering and/or glazing significantly improved the marginal fit.

## 1. Introduction

A growing number of patients are requesting esthetic restorations, which has led to the development of all-ceramic restorations for teeth in the anterior esthetic zone as well as for posterior teeth. Despite the fact that conventional metal–ceramic restorations offer reasonably good esthetics, knowing that gingival tissues are transparent, they may affect the color of the free gingival margin [1]. Because of the opacity of the metal base, the entire restoration loses its translucent aspect [2,3]. A large number of modern all-ceramic restorations show outstanding physical and mechanical characteristics, which makes them very desirable [4,5]. Zirconia ceramics, mainly 3Y-TZP, are getting more popular as a suitable material for posterior teeth restoration and is frequently chosen over other ceramic materials [6,7]. 3Y-TZP is currently the ceramic material available with the highest mechanical properties as compared to its other ceramic counterparts; however, its major drawback is that its translucency seems to be much lower than that of glass-based ceramics, which show high esthetic value [8].

Lithium disilicate (LD) exhibits greater translucency but less mechanical strength than zirconia [9,10]. Ceramic restorations can be created using a variety of manufacturing procedures, such as the computer-aided design and manufacturing (CAD/CAM) technique. It is important to note that monolithic and layered approaches can be used to produce zirconia and LD restorations. The development of CAD/CAM, which was introduced into dentistry more than 20 years ago, is directly tied to the industrialization of zirconia [11,12,13]. By using commercially manufactured blocks of material, CAD/CAM systems can produce restorations of greater and consistent quality. They can also regulate restorative shaping processes, resulting in lower manufacturing costs and shorter processing times [14,15].

There are certain disadvantages to using CAD/CAM technologies as well. The scanning mechanism is limited by its restricted resolution, which can result in margins that are moderately rounded [15]. Some of the intrinsic imperfections in CAD software algorithms used to turn point clouds into a smooth and continuous surface can also contribute to interfering contacts at the incisal/occlusal edge, which are demonstrated to be deleterious if they occur near the margin [16]. Different cervical margin designs and ceramic manufacturing techniques have been investigated for all-ceramic crowns [17]. In addition, the ceramic firing cycles and glaze firing cycles have shown effects on the marginal fit of all-ceramic restorations [18]. The veneering process, which includes a firing procedure (sintering) at high temperature and subsequent cooling of the restoration, is carried out at least once, but usually 2–5 times [19].

Marginal and internal accuracy of fit is valued as one of the most important criteria for the clinical quality and success of all-ceramic crowns [20]. In fact, the marginal fit has a variety of profound consequences, some of which may result in the prosthesis malfunctioning unexpectedly. Large marginal inconsistencies lead to thick cement/luting agent layers subjected to higher dissolution or degradation by the aggressive oral environment as a result of oral fluids and chemo-mechanical pressures [21,22,23]. The cement barrier becomes brittle, allowing bacteria to penetrate the tooth and perhaps causing inflammation of the vital pulp. One in vivo study showed that high marginal discrepancies in fixed prosthetic restorations are associated with greater plaque index, lower periodontal health, and secondary caries [22]. Due to the huge margin of error associated with most of the used methods, some studies used the absolute marginal discrepancy which is an angular combination of horizontal and vertical marginal gaps [21]. This measurement shows the total mismatch at the margin and is always considered the largest measurement of the inaccuracy at that point in the process [24]. Other methods described the horizontal and vertical gaps as the two measurements depend on the point angle of two tangents as a reference point for both measurements [22,25].

Although previous studies have evaluated marginal fit of zirconia and lithium disilicate restorations, limited evidence exists on the combined effect of material type, restoration design, and post-processing (veneering/glazing) within a single standardized experimental model. Furthermore, the influence of multiple firing cycles on marginal adaptation across different CAD/CAM materials remains unclear.

Zirconia and lithium disilicate restorations were evaluated in this in vitro study for their marginal fit after two veneering techniques (veneering or glazing), as well as an evaluation of whether the glazing affects the marginal fit. The null hypothesis was that marginal fit would not be significantly affected by (1) material (zirconia vs. lithium disilicate), (2) restoration design (monolithic vs. coping), or (3) finishing protocol (as-produced vs. veneered/glazed), nor by their interactions.

## 2. Materials and Methods

**Figure 1:**
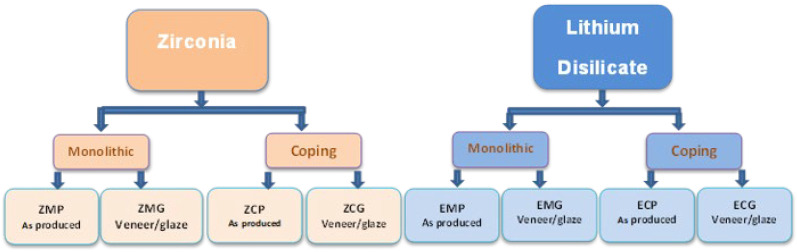
Experimental design (n = 8) and 8 different groups.

The experimental design of the study is illustrated in Figure 1. The crowns were produced using CAD/CAM technique from two different types of materials: 4Yzirconia (Zenostar^®^ (Z) and lithium disilicate (IPS e.max^®^ CAD (C) (Ivoclar Vivadent, AG, Schaan, Liechtenstein) both A2 shade. Sixteen crowns were separated into monolithic (M) and core (C). Furthermore, every group was split into a second subgroup, which involved glazed/veneer (G) and as-produced without glazing (P). The following groups were generated by using all possible combinations to create eight different groups: ZMP, ZMG, ZCP, ZCG, EMP, EMG, ECP, and ECG. There were eight repetitions (n = 8) for each unit, resulting in a total of 64 restorations. Sample size was determined based on our pilot study evaluating marginal discrepancies in CAD/CAM restorations. A minimum of n = 8 specimens per group was considered sufficient to detect a clinically relevant effect size of approximately 15–20 µm with a power of 80% and α = 0.05. This corresponds to a moderate to large effect based on previously reported marginal gap variability in CAD/CAM restorations.

### 2.1. Master Die

**Figure 2:**
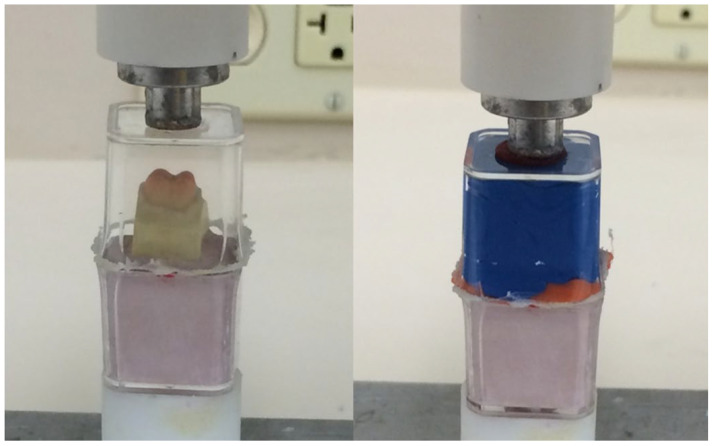
Custom-made alignment device.

A master mandibular model of tooth # 30 with manufacturer preparation for all-ceramic crowns from Ivoclar Vivadent was used in this study. (Reorder #594148) The preparation (1.6 mm occlusally and 1.4 mm axially) with 1.4 mm deep shoulder margin was used as the master model for all crowns. As a first step, the master model was duplicated to obtain 64 plaster models made of hard die stone (Violet Stone type 4 Silky-Rock, WhipMix Corporation, Louisville, KY, USA). Using a glass plate, wax, and a custom-made surveyor, the model was oriented perpendicular to the table surface. A custom-made alignment device was then used to center the die into a custom-made 2 × 2 × 2 cm plastic container, which was filled with Violet Stone. Then, using the same alignment device and a custom-made, standardized impression tray, along with polyvinyl siloxane impression material of both light and heavy consistency (Virtual XD, Ivoclar Vivadent, Amherst, NY, USA), an impression was taken with an equal thickness of impression material. The impression was subsequently poured using violet Stone. Using this approach made it possible to achieve an exact position of each stone model relative to a standard reference plane (Figure 2).

### 2.2. Manufacturing of the Crowns

**Figure 3:**
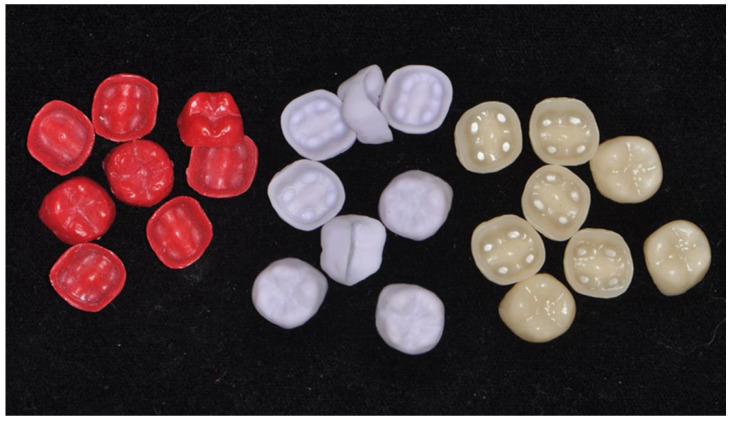
Lithium disilicate wax patterns, cores and veneered crowns.

To produce standardized thickness for both materials, wax patterns had to be produced and scanned. To create the wax build-up for the cores and monolithic crowns, a wax injection device (Rio Grande, Albuquerque, NM, USA) and red wax (Jewelry injection wax, West cast, Albuquerque, NM, USA) were used. For the monolithic crowns, an injection hose mold was designed that accommodated the wax injector and to be adjusted on the die to standardize the wax thickness 1.4 mm for the monolithic crowns and 1 mm for the copings. Lithium disilicate crowns were fabricated with the CEREC System (CEREC 3D Blue Cam, Dentsply Sirona, Charlotte, NC, USA). An optical impression was taken of the master die and 16 monolithic crowns were milled (Figure 3). The 16 lithium disilicate cores were milled using the copy anatomy option in CEREC software version 5.3 from the scanned wax patterns as was done for the crowns.

For the zirconia fabrication, a 3Shape E4 scanner (3shape A/S, Copenhagen, Denmark was used to scan both the master die and master wax patterns, and designed using the 3Shape dental system. Zirconia was milled from Zenostar disks by the laboratory of Ivoclar Vivadent North America (Zenotec Mini, Wieland, IL, USA) and sintered using a Wieland sintering furnace with the standard 1450 °C/2 h program (Table 1). A total of 16 identical monolithic crowns were milled. The external surface of the crown was polished with ProArt^®^ polishing wheels (Ivoclar Vivadent AG, Schaan, Liechtenstein) using 15k rpm with Zenostar polishing paste (Zenostar polish, Wieland, IL, USA). The Z cores were fabricated in the same manner, depending on the scanned core’s wax pattern. Using this construction, 16 identical cores were milled using the Zenotec milling machine (Zenotec; Technik GmbH & Co., Pforzheim, Germany). Crowns and cores of groups ZMG, ZCG, EMG, and ECG were either glazed or veneered and glazed in the manner prescribed below.

Zirconia coping (ZCG): Using a paintbrush, a thin layer of ZirLiner (IPS e.max ZirLiner Ivoclar Vivadent) was applied before the fire process according to the manufacturer’s firing parameters (Table 2).

Following that, one layer of IPS e.max^®^ Ceram dentin was mounted directly to 0.5 mm short of the margin over the cores to shape and contour the crowns, and then they were fired using the settings described in Table 3. Lastly, a thin layer of IPS e.max^®^ Glaze powder and liquid was applied to the veneered crowns, which were then heated according to the specifications in Table 3 to complete the process. A thin layer of e.max Glaze powder and liquid was applied to the zirconia monolithic crowns (ZMG) in (Table 2): zirconia monolithic crowns (ZMG) were glazed by a thin layer of e.max Glaze powder and liquid.

Cores IPS e.max CAD (ECG) following crystallization were veneered as follows: a single layer of IPS e.max Ceram dentin was applied 0.5 mm short of the margin over the cores to shape and contour the crowns and fired according to the specifications listed in Table 4. The next phase in the process was to apply a thin layer of IPS e.max Glaze powder and liquid to the veneered crowns, which were then heated according to the parameters stated in Table 4. Lithium disilicate monolithic crowns (EMG) were crystallized and glazed with a thin layer of e.max Glaze powder and liquid. The crowns and cores of groups ZMP, ZCP, EMP, and ECP were used as-produced.

### 2.3. Cementing of the Crowns and Cores

The inner surfaces of LD groups were treated with 5% hydrofluoric acid (IPS Ceramic Etching Gel, Ivoclar Vivadent) for 20 s. The intaglio surface of zirconia restorations were lightly air abraded using less than 1 bar (14.5 psi) with 100 micron alumina particles. All groups were treated with a thin coat of conditioner (Monobond Plus, Ivoclar Vivadent) and the solvent was evaporated for 1 min prior to applying the resin cement. To ensure consistency of the cementation procedure, a standardized amount of resin cement was applied to the intaglio surface using an automix syringe tip (Multilink Automix, Ivoclar Vivadent). A thin uniform layer of stain (Tetric color, Ivoclar Vivadent) was incorporated at approximately 5% by volume to enhance visualization. Excess cement was removed prior to polymerization under constant seating load. For load standardization, all crowns were inserted in the same perpendicular path and seated at a load of 70 newtons with a custom-designed device to simulate clinical finger pressure during cementation, consistent with previous in vitro studies evaluating crown seating forces [22,25]. Light curing was performed for 20 s per surface (occlusal, mesial, distal, buccal, and lingual) using a calibrated LED curing unit VALO Grand (Ultradent Products, Inc., South Jordan, UT, USA) The light tip was positioned approximately 1 mm from the crown surface using a custom positioning guide to ensure consistent distance and angle across all specimens. Radiant exposure was standardized at 10.79 J/cm^2^ for all samples.

### 2.4. Embedding and Sectioning of the Cemented Crowns

**Figure 4:**
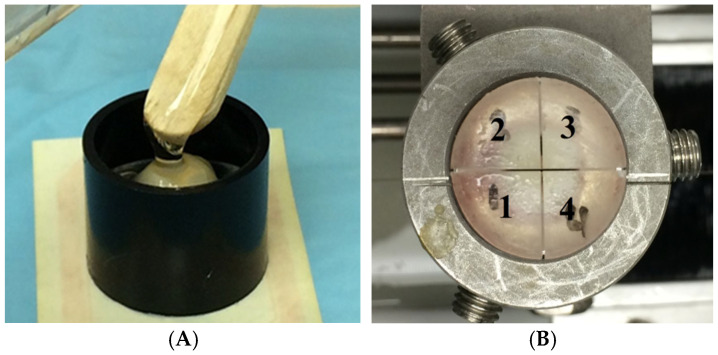
(**A**). Embedding cemented crown with transparent epoxy resin. (**B**). Sectioning the cemented crown. The numbers refer to the sectioned quadrants order.

A standardized mold was used to fix the crowns into epoxy resin (EpoFix kit, Struers, Ballerup, Denmark) (Figure 4A). This mold can be fitted into the sectioning device Buehler Isomet 1000 (Buehler, Ltd., Lake Bluff, IL, USA). The unit was cut in two perpendicular directions from the occlusal surface—buccal–lingual and mesial–distal—in the midpoint of each side using the same cutting speed and weight (150 rpm,150 g). As a result, the marginal fit of the crown was evaluated by using 4 sections (Figure 4B).

### 2.5. Measuring the Gaps

**Figure 5:**
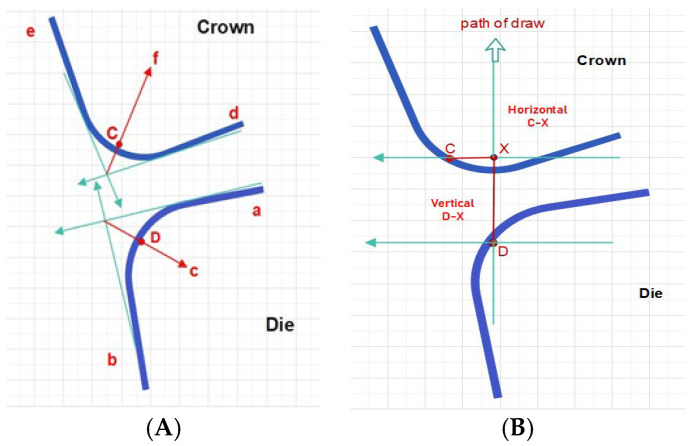
(**A**,**B**): Horizontal and vertical measurements.

**Figure 6:**
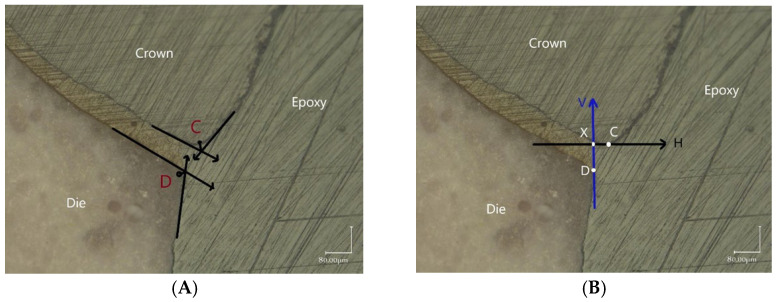
(**A**,**B**): Vertical and horizontal point definitions for the evaluation of horizontal and vertical gap.

Selected points in all sections were measured on the margin at a magnification of 200× to obtain the marginal gap using a digital microscope Keyence VHX-1000 (Keyence Corporation, Osaka, Japan). The criteria as described by Holmes et al. had to be modified due to the slightly rounded edges that were found on both the ceramic and the die, as reported in an earlier study by Abdulhameed et al. [25]. The technique is illustrated in Figure 5A. To begin, a straight line (a) is drawn from the prepared shoulder, and a second line (b) is drawn from the die’s outer surface. Subsequently, the angle bisector line (a + b) is drawn (c). The region where the angle bisector line intersects the die is known as the measuring point D. Two lines are drawn to select an analog point on the restoration margin (C): one is the straight extension of the restoration’s inner surface (d), and the other is the straight extension of the restoration’s outer surface (e). At the junction of the angle bisector path (f) and the restoration, measuring point C is established. To put it another way, the vertical misfit is the vertical distance between two intersecting horizontal lines, D and C. To measure horizontal misfit, the distance between C and the crossing (X) of D’s path is measured on a horizontal line (Figure 5B). Figure 6 is an example of the application under microscope. The measurements were performed for every trial group to measure the mean values alongside the standard deviation of gap measurements. The reference points (C and D) were defined based on the modified protocol described by Holmes et al. [20] and Abdulhameed et al. [25], using tangent lines and angle bisectors to standardize measurement in rounded ceramic margins.

### 2.6. Statistical Analysis

Statistical analysis was performed using SPSS Statistics for Windows, Version 28.0 (IBM Corp., Armonk, NY, USA). Data normality was assessed using the Shapiro–Wilk test. Homogeneity of variances was verified using Levene’s test (*p* > 0.05), confirming suitability for parametric analysis. Descriptive statistics (mean ± standard deviation) were calculated. A three-way ANOVA was initially conducted to evaluate the effects of material, design, and finish, followed by two-way ANOVA within each material. Post hoc pairwise comparisons were performed using Tukey’s HSD test. Statistical significance was set at α = 0.05.

## 3. Results

**Figure 7:**
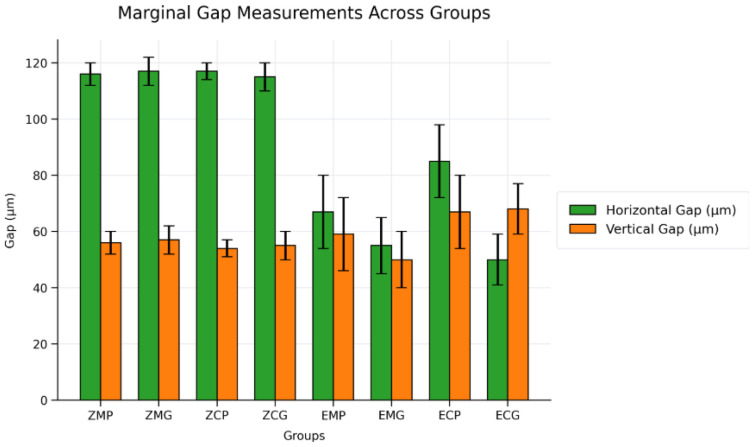
Mean horizontal and vertical marginal gaps (µm) across experimental groups (ZMP, ZMG, ZCP, ZCG, EMP, EMG, ECP, ECG). Error bars represent standard deviations (±SD).

The measurements were performed for the Horizontal and Vertical distance four times per sample and the mean for each group was reported separately. A two-way ANOVA was conducted separately for each material to evaluate the effects of design (monolithic vs. coping) and finishing protocol (as-produced vs. veneered/glazed). For zirconia, neither factor nor their interaction showed statistical significance (*p* > 0.05). For lithium disilicate, the finishing protocol showed a statistically significant effect (*p* < 0.05), while neither the design factor (*p* > 0.05) nor the interaction between design and finishing (*p* > 0.05) reached statistical significance. Post hoc Tukey tests confirmed that EMG and ECG had significantly smaller horizontal gaps compared to EMP and ECP. The average horizontal gap between the shoulder lines was significant (*p* < 0.05): 116 ± 5 µm in the Z groups and 64 ± 13 µm in the LD groups. No statistically significant differences were found among zirconia groups (ZMP, ZMG, ZCP, ZCG) (*p* > 0.05, ANOVA), despite minor numerical differences. No statistically significant differences were found among zirconia groups (*p* > 0.05; η_p_^2^ < 0.10), indicating a small effect size. See Figure 7 for means and ±SD.

However, there were no significant differences in the vertical gap for all groups, with values of 55 ± 4 µm for the ZR group and 61 ± 7 µm for the LD group. The results showed that multiple firings have no influence on the marginal fit for all Zr groups. The horizontal gaps were approximately twice as big as the vertical gaps for these groups. The data collected from LD groups showed that the horizontal gaps were approximately half the size of the Zr ones. Since the horizontal gap is considered more clinically significant than the vertical gap [26], we created an index to make the results more clinically relevant. We were weighing the horizontal gap ×2 and the vertical ×1 to see the overall effect of material, design, and finishing on the marginal discrepancy. Table 5, which presents the clinical significance index, showed that LD has a lower index overall than the Zr groups. Within LD groups, the monolithic glazed had the lowest index (Table 6), followed by the coping with the veneered/glazed group. The finishing protocol showed a statistically significant effect (*p* < 0.05; η_p_^2^ ≈ 0.35), indicating a large effect size. All groups for both materials showed marginal gaps within acceptable limits, below 120 µm, according to Holmes et al. [20].

## 4. Discussion

The first study, which determined an obvious definition for the points utilized to measure vertical and horizontal gaps for porcelain fused to metal (PFM) crowns, was Holmes et al. [20]. In this study, internal gaps and marginal discrepancies were distinguished. Though when working with PFM crowns, the edges of the metal were sharp, and the crown preparations were incredibly sharp as well, compared to all-ceramic preparations. For this reason, the landmarks were used to establish the measurement points. Before doing this trial, an earlier study [25] had discovered that when using ceramic crowns, there were no sharp features on the crowns or on the crown preparations, making it impossible to locate the features on a microscopic scale. The high SD caused the result of the measurement to be unconvincing. It was decided to utilize tangents and bisecting angle lines to create well-defined, detailed, and reliable measuring sites, which resulted in a low SD for the marginal gaps. In terms of determining internal gaps, Holmes et al. [20] used the occlusal and axial gap concepts, then modified them by pinpointing the exact location and taking measurements at several areas. The commonly utilized zirconia and lithium disilicate crown/coping were used in order to compare various manufacturing techniques with similar materials. Zenostar and e.max CAD was the material of use. The quantity of area and the number of measuring sites in this study were limiting factors. To obtain these precise measurements, a single measuring point was used (horizontal and vertical) for every quadrant, with a total of eight points per crown. This method ensured that the horizontal and vertical reference points were correctly chosen, enhancing the significance of these measurements. Each group had a total of 128 points of measurement. Once divided into four blocks, eight functional surfaces are produced for calculations. A 0.5 mm thick diamond blade (the same blade) was used to get equivalent measurements for both as indicated by the preliminary study, and it was planned, due to their proximity, to only analyze one surface of each cut. This study assessed the marginal fit of two clinically commonly used CAD/CAM anatomic contour 4Y zirconia crowns (ZENOSTAR translucent of Wieland Dental) and lithium disilicate glass ceramic crowns (IPS e. max CAD of Ivoclar Vivadent). To replicate clinical conditions, an acrylic resin tooth was used in this trial. Surveyor milling machines were used to prepare the tooth in a predictable way. Every crown was dissected in the same place, with four guideposts. Using acrylic teeth instead of natural teeth isolates confounding factors derived from dentin special characteristics. Although humidity, resilience and substrate behavior are different between acrylic and natural teeth, in this experiment, where only a marginal gap is measured, this methodological choice is adapted by other authors [27,28]. To model the clinical environment during cement setting, the crowns were cemented under constant weight of 68.6 N which was based on a clinical simulation, and ten minutes of finger pressure was applied to each of the crowns. LD specimens were treated with 5% hydrofluoric acid for 20 s while Zr specimens were lightly air abraded using less than 1 bar with 100 um alumina particles. The selection of conditioner (Monobond Plus, Ivoclar Vivadent) and Multilink Automix resin cement ensure high bond strengths in both zirconia and LD substrates [29,30]. The cementation protocol is another aspect that deserves further consideration. The measurement of marginal fit is often done by either sectioning embedded materials [22,24,31] or directly visualizing them [32,33]. One of the benefits of direct visualization is that it can measure without damaging specimens, making it particularly useful in clinical situations. However, it is unfortunately difficult to obtain accurate readings by simply looking at the object [24]. In this study, the sectioning approach was employed in order to obtain accurate measurements of marginal fit. When zirconia was utilized on a preparation with a shoulder margin, the results showed that the maximum gap at the margin was 118.8 µm. It is foundational for fixed prosthodontics that different finish lines and other factors influence marginal adaptation [17]. The preparation of the deep shoulder margin followed in this experiment is less favorable for marginal adaptation since a significant bulk of restorative material is required [3,17]. According to most researchers, the proportion of the marginal gap was within the clinically acceptable limits. By using the CAD/CAM system, the marginal fit of restorations created was optimized. The CAD/CAM technology eliminated handwork errors; however, other procedures were added to computers, resulting in some defects [34]. Given the increasing material volume of anatomic zirconia crowns compared to zirconia copings, the shrinkage error could arise before and after sintering, resulting in a bigger marginal gap compared to earlier findings. It is well-established knowledge that several factors influence the internal and marginal fit of crowns and FPDs [35]. Our results in lithium disilicate crowns are similar to Shadur et al. [36] and zirconia crowns similar to Fasih et al. [37].

In the results, we used an index to weigh the harmful effects of any discrepancies and make the data clearer to clinicians. The weighting was based on clinical experience [38]. From a clinical standpoint, a slight vertical discrepancy will result in a thicker cement layer at the margin, which can be difficult to manage (×1). However, horizontal discrepancy provides an overhang creating a plaque accumulation zone, which is considered harmful with regard to its potential to induce gingivitis, periodontitis, and recurrent caries [22,26]. Therefore, the highest factor (×2) was assigned to the horizontal gap. Internal gaps are of less significance since they determine the cement layer thickness. Due to the determined internal gap size (die spacer or cement thickness in CAD/CAM), we did not find “negative gaps,” e.g., direct contact with the die. Therefore, we decided not to evaluate the internal gaps. Validation of this factor is only possible with another clinical study, which was not the scope of the present paper.

The clinical significance index proposed in this study provides a simplified method to integrate horizontal and vertical discrepancies into a single value. However, this index has not been independently validated and should be interpreted cautiously. Previous studies have assessed marginal fit using separate measurements or absolute marginal discrepancy values [21,24]. Future research should validate this index against clinical outcomes.

The variability in marginal fit between systems is caused by zirconia blocks with different shrinkage levels which depend on the production line, scanning methodology, milling bur size, and milling axis count. This analysis has some drawbacks. The clinical finger pressure used to bond crowns onto stone dies was not defined in previous studies. Therefore, a loading mechanism was used to provide detailed requirements for each crown, which was standardized. Furthermore, stone dies were used instead of teeth to assess the marginal fit of anatomic contouring crowns. A human tooth would be perfect for modeling a clinical scenario, even if stone dies or resin dies have been previously accepted [32,38].

The findings of this trial indicate that lithium disilicate CAD is superior to zirconia when manufacturing an anatomic contour crown in terms of the marginal gap. To accommodate for the possible shrinkage of zirconia during fabrication, more research and clinical applications are needed. Precision milling requires improvements in CAD/CAM technology [27]. Within the limitations of the present study and the specific veneering/glazing protocols used, multiple firing cycles did not significantly influence marginal fit in zirconia groups.

Additionally, glazed restorations had a smaller marginal gap when compared to unglazed restorations in LD crowns. Therefore, further studies are necessary to investigate the effect of glaze on the marginal fit of LD restorations. Manufacturer recommendations should always be followed when glazing all-ceramic restorations. Although marginal fit is undoubtedly an important parameter, other clinically relevant properties should also be considered when discussing the best restorative approach. However, the results of this study stay focused on manufacturing and material factors with a potential to influence marginal fit.

This in vitro study used stone dies and standardized conditions that may not fully replicate intraoral variability. Different CAD/CAM workflows were used for lithium disilicate and zirconia restorations (CEREC System for LD and 3Shape/Wieland workflow for zirconia). The differences observed may not be exclusively related to the restorative material itself, but also to the scanner, software, milling strategy, or manufacturing process. The loading protocol and firing cycles were limited to specific systems. The study employed a controlled factorial design evaluating three independent variables with standardized measurement methodology.

Future studies should investigate long-term clinical outcomes, the effect of different CAD/CAM systems, and validate the proposed clinical significance index in vivo. Clinical studies performed with this index as a landmark for long-term clinical performance of all-ceramic restoration will be evaluated with other clinical measurements. In light of the above, in the aggressive oral environment, these differences found in this study should be reconsidered for conclusive results.

## 5. Conclusions

Within the limitations of this in vitro study, all groups demonstrated horizontal and vertical marginal discrepancies below 120 µm, which are considered clinically acceptable.

The null hypothesis was partially rejected, as marginal fit was significantly influenced by material type and finishing protocol, while restoration design showed no consistent effect. Lithium disilicate restorations exhibited significantly smaller horizontal marginal gaps compared to zirconia. In addition, finishing procedures (veneering/glazing) significantly improved the marginal fit of lithium disilicate restorations, whereas no significant effect was observed in zirconia groups.

Among all groups, the glazed monolithic lithium disilicate crowns (EMG) and veneered/glazed lithium disilicate copings (ECG) demonstrated the smallest marginal discrepancies. In contrast, zirconia groups showed consistently larger marginal gaps regardless of design or finishing protocol.

## Figures and Tables

**Table 1:** Sintering settings for zirconia specimens (cores and monolithics).

	Temperature 1 [°C]/[°F]	Temperature 2 [°C]/[°F]	Heating Rate [°C/h]/[°F/h]	Holding Time [h]
Heating phase	20/68	900/1652	600/1112	-
Holding phase	900/1652	900/1652	-	0.5
Heating phase	900/1652	1450/2642	500/932	-
Holding phase	1450/2642	1450/2642	-	2
Cooling phase	1450/2642	900/1652	600/1112	-
Cooling phase	900/1652	300/572	500/932	-

**Table 2:** Firing parameters for layering ZCG crowns. B (°C): Start temp, S (min): Dry time, t (°C/min): Heating rate, T (°C): Peak temp, H (min): Hold time, V1 (°C): Vacuum on, V2 (°C): Vacuum off.

IPS e.max Cream	B °C	S min	t °C/min	T °C	H min	V1 °C	V2 °C	L °C
Zirliner firing	403	4:00	40	960	1:00	450	959	0
Wash firing	403	4:00	40	750	1:00	450	749	0
Dentin-/incisal firing	403	4:00	40	750	1:00	450	794	0
Glazing firing	403	4:00	60	750	1:00	450	724	450

**Table 3:** Firing parameters for staining ZMG crowns. B (°C): Start temp, S (min): Dry time, t (°C/min): Heating rate, T (°C): Peak temp, H (min): Hold time, V1 (°C): Vacuum on, V2 (°C): Vacuum off.

IPS e.max Ceram	B °C	S min	t °C/min	T °C	H min	V1 °C	V2 °C
Stain and characterization firing	403	6:00	60	770	1:00	450	769
Glaze firing	403	6:00	60	770	1:00–2:00	450	769
Add-on after glaze firing	403	6:00	50	770	1:00	450	699

**Table 4:** Firing parameters for veneering and glazing of IPS e.max CAD. The arrow indicates an ascending temperature. B [°C/°F]: Start temp, S Dry time, t↗ [°C/°F/min]: Heating rate, T [°C/°F]: Peak temp, H Hold time, V1 [°C/°F]: Vacuum on, V2 [°C/°F]: Vacuum off.

IPS e.max Ceram on IPS e.max CAD Layering Technique	B [°C/°F]	S [min]	t↗ [°C/°F/min]	T [°C/°F]	H [min]	V1 [°C/°F]	V2 [°C/°F]
Dentin and incisal firing	403/757	4:00	50/90	750/1382	1:00	450/842	749/1380
Glazing firing	403/757	6:00	60/108	725/1337	1:00	450/842	724/1335

**Table 5:** Gap location data. The mean of the gap measured in µm for Zr and LD. No statistically significant differences for same letters. D is the smallest horizontal gap and d is the smallest vertical gap. Upper case letters for Horizontal gab and lower case letters for vertical gap. E = best, A = worst.

Gap Location	Zenostar				e.max CAD				Statistic
	Monolithic		Coping		Monolithic		Coping		
	ZMP	ZMG	ZCP	ZCG	EMP	EMG	ECP	ECG	
Horizontal	116 A	117 A	117 A	115 A	67 C	55 D	85 B	50 D	*p* < 0.001
Vertical	56 d	57 d	54 d	55 d	59 d	50 d	67 c	68 c	*p* < 0.001

**Table 6:** Accumulated index and ranking. Same letters indicate no significant differences (*p* < 0.05). E = best, A = worst.

Gap Location	Zenostar				e.max CAD			
	Monolithic		Coping		Monolithic		Coping	
	ZMP	ZMG	ZCP	ZCG	EMP	EMG	ECP	ECG
Horizontal ×2	232	234	232	230	134	110	170	100
Vertical ×1	56	57	54	55	59	50	67	68
Accumulated Index	288	291	288	285	193	160	237	168
Ranking	A	A	A	A	C	E	B	D

## Data Availability

The original data supporting the findings of this study are stored securely by the authors and are available upon reasonable request. Further inquiries can be directed to the corresponding author.
